# Motion analysis and structural parameter optimization for nested multi-link transplanting mechanism of variable plant spacing transplanting machine

**DOI:** 10.1371/journal.pone.0337811

**Published:** 2025-12-04

**Authors:** Jian Kang, Subo Tian, Siyao Liu, Jianbo Liu, Yabin Fu, Hejin Wang, Muhammad Awais

**Affiliations:** 1 College of Engineering, Shenyang Agricultural University, Shenyang, China; 2 Key Laboratory of Facility Horticulture in Ministry of Education, Shenyang Agricultural University, Shenyang, China; 3 College of Horticulture, Shenyang Agricultural University, Shenyang, China; 4 Baoji Ding duo Machinery Co. Ltd, Baoji, China; National Institute of Agricultural Research - INRA, MOROCCO

## Abstract

The nested multi-link transplanting mechanism features a compact structure suitable for transplanting machines and enables zero-speed transplanting, which is crucial for high-quality seedling establishment. To address the challenge of existing transplanting mechanisms being unable to ensure the transplanting effect when the plant spacing changes, this study investigated the influence of key structural parameters of nested multi-link mechanisms on the motion characteristics and planting performance of transplanting devices. First, we derived the kinematic model of the nested multi-link transplanting mechanism and developed a MATLAB/GUI interface to simulate end-effector trajectories. We then quantified how individual parameters affect both the trajectory and the tip velocity of the planting end-effector. Third, we formulated a multi-objective optimization problem to maintain consistent planting depth, seedling uprightness, and adequate trajectory height across three spacing settings (350, 400 and 450 mm) and solved it with a multi-objective genetic algorithm (MOGA) to obtain Pareto-optimal structural parameters. Finally, to validate the proposed model, we compared the simulated end-effector trajectories with high-speed video data collected from a physical prototype. Simulations reveal that adjusting only the vertical position of the power-transmission pivot (point B) is sufficient to maintain transplant quality across all target spacings. Field tests at 400 mm spacing yielded a 98% qualified-planting rate with an improvement of 3.56 percentage points higher than the pre-optimization baseline. Additionally, the lodging planting rate was 1.11%, the missing planting rate was 0.22%, the coefficient of variation for plant spacing was 3.01%, and the planting depth qualification rate was 95.11%, all meeting transplanting standard requirements. These findings demonstrate that the proposed MOGA-based parameter calibration consistently achieves high transplant quality under variable spacing conditions, thereby providing a practical design framework for precision vegetable transplanters., and thus provides valuable insights for improving nested multi-link transplanting mechanisms and advancing mechanized vegetable cultivation.

## Introduction

Seedling nursery and transplanting technologies offer substantial advantages in agricultural production including shortened crop growth period, the mitigation of seasonal and climatic constraints [1], reduced risk from natural disasters, and improved seedling survival rates. As a result, it has been widely adopted as a standard practice in vegetable production systems worldwide [2,3]. However, a common challenge persists: conventional transplanters often generate inconsistent planting trajectories under varying plant-spacing settings. These fluctuations in instantaneous velocity and acceleration disrupts the essential zero-speed planting condition, and leading to frequent seedling lodging and reduced transplanting quality, which ultimately diminishes crop yield [4]. As the core component of the transplanter, the planting mechanism governs these the motion characteristics and therefore directly affect the quality and success of vegetable seedling transplanting [5,6].

According to the mechanical structure and working principle of the planting mechanism, planting mechanisms can be categorized into flexible disc type [7,8], chain-clip type [9,10], cup-type [11–14], duckbill type [15], and seedling guide tube type [16]. Among these, the flexible disc, chain-clip,and seedling guide tube type requires an additional hole opener to place seedlings into pre-formed holes [17], making them unsuitable for direct vegetable transplanting where pot seedlings must be inserted straight into the soil. In contrast, the duckbill type typically employs multi-link mechanisms [18], which provide considerable design flexibility and can achieve zero-speed transplanting a critical capability for enhancing seedling uprightness. By adjusting structural parameters, multi-link mechanisms can generate diverse planting trajectories and motion speeds, thereby accommodating the specific needs of various crops [19].

Previous studies have explored multi-link mechanisms for transplanting applications. Sharma et al. [20] developed a five-bar duckbill planting mechanism, established kinematic equations, and designed the planting mechanism parameters according to agronomic requirements. However, the mechanism’s fixed structure limits its adaptability to other planting parameters. Similarly, Jin et al. [21] designed a five-bar duckbill planting mechanism that improved transplanting quality under specific conditions, but performance deteriorated with changes in planting parameters. Collectively, these studies show that multi-link architectures improve compactness and adaptability, yet zero-speed planting is only maintained through exact kinematic calibration.

To achieve zero-speed transplanting, several approaches have been proposed. Wen et al. [22] adjusted the transmission ratio so that the duckbill tip velocity at the trajectory bottom exactly opposed the machine’s forward speed. Xiao et al. [23] ensured the horizontal velocity below ridge contour line was near zero, thereby improving seedling uprightness. Na et al. [24] identified zero-speed points through velocity analysis to meet planting requirements. These studies confirm that multi-link mechanisms can fulfill the requirement for compact and adjustable transplanting systems. However, systematic parameter optimization is required to maintain performance when plant spacing changes. Specifically, key indicators like consistent planting depth and seedling uprightness cannot be guaranteed without such optimization.

Researchers have optimized multi-link mechanisms via virtual prototyping and mathematical programming. He et al. [25] used a human-machine visual interface to calibrate the 2ZBX-2A vegetable transplanter, achieving upright seedlings and minimal film damage, yet they tested only a single plant spacing. Ali et al. [26] developed a bar-type planting mechanism and optimized its trajectory, which raised the success rate, but seedling uprightness remained suboptimal. A key limitation of these studies is their exclusive focus on optimizing parameters for a single planting spacing. Since vegetable cultivation requires variable spacings (e.g., 350–450 mm) to meet diverse agronomic and regional demands, a mechanism adaptable of adapting to multiple configurations are essential.

To overcome these limitations, we present an adjustable nested multi-link mechanism designed to maintain optimal transplanting performance across different plant spacings. Unlike conventional multi-link systems, our nested linkage retains zero-speed planting by simply adjusting the vertical position of the fixed pivot (point B); this single parameter compensates for spacing changes without re-tuning the entire linkage. While traditional optimization methods, such as analytical approaches or trial-and-error and single-objective strategies, often struggle with the high-dimensional and strongly coupled constraints inherent in such complex mechanisms, We employed a multi-objective genetic algorithm (MOGA) to systematically determine the optimal structural parameters under high-dimensional, strongly coupled constraints. The algorithm simultaneously satisfied three key agronomic requirements: planting loop depth, planting loop perpendicularity, and total trajectory height, thus enabling an automated and globally optimal design. The subsequent sections cover the development of a kinematic model, analysis of critical parameter effects, optimization for target spacings, and validation via simulation and experiment.

## Materials and methods

### Working principle of transplanting machine and transplanting mechanism

**Fig 1:**
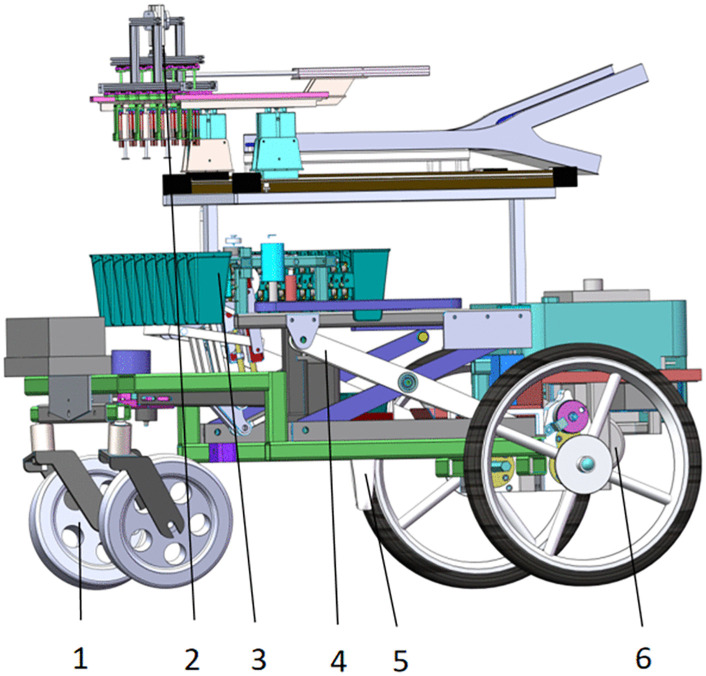
Transplanting machine chassis structure diagram. 1. Guide wheel; 2. Seedling pick-and-place device; 3. Seedling cup; 4. Lifting mechanism; 5. Planting mechanism; 6. Drive motor.

This study focuses on vegetable transplanting in the dryland or sandy soils of south-western China, where 350–450 mm plant spacing is commonly recommended to ensure adequate ventilation, light penetration and seedling survival. Therefore, the transplanting machine shown in Fig 1 was selected as research subject for this study.The transplanting machine mainly consists of three parts, the walking part, the seedling picking part, and the planting part.

The transplanter is propelled by its rear driving wheels. Its key functional components. the planting mechanism and the seedling pick-and-place device, are all driven by individual motors. The working process of the transplanting machine is as follows. First, the seedling pick-and-place device extracts seedlings from the trays and deposits them into the rotating seedling cup. This cup then transports the seedling to the planting mechanism, which finally transplants the seedlings into the ridges.

This system executes a completes operational cycle for seedling transplanting from picking to final placement into the soil. Since all the power units are motors, enabling independent control over the seedling pick and place rate, the rotation speed of the seedling cup, the planting frequency, and the forward velocity. A scissor-lifting mechanism is welded onto the chassis frame, and provides adjustment of the planting depth. When the duckbill planter reaches a certain depth, the duckbill of the planting mechanism opens through a pull wire mechanism, and closes completely after it is higher than the seedling elevation. Furthermore, the planting spacing can also be adjusted by changing the forward speed of the machine. Although the planting spacing and the planting depth can be adjusted mechanically, seedling uprightness and other quality metrics deteriorate if linkage dimensions are not properly tuned.

Therefore, this study mainly focuses on optimizing the motion trajectory and key structural parameters of the transplanting mechanism to ensure that the transplanting machine can still achieve the optimal planting effect after adjusting the plant spacing.

### Kinematic analysis of the transplanting mechanism

**Fig 2:**
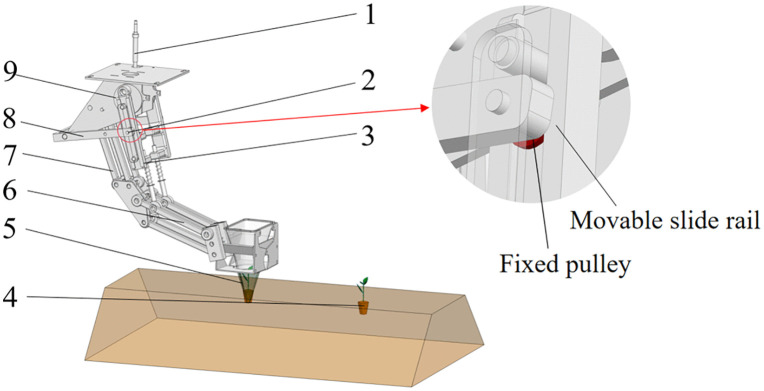
Structural diagram of planting mechanism. 1. Planting trajectory adjustment device; 2. Rotation center of power transmission rod; 3. Sliding rod; 4. Seedling; 5. Duckbill planting end effector; 6. Parallel four-bar mechanism I; 7. Parallel four-bar mechanism Ⅱ; 8. Adjustment rod; 9. Drive wheel.

As shown in Fig 2, The nested multi-link transplanting mechanism has one degree of freedom (DOF). A drive wheel actuates a sliding rod, which subsequently drives the parallel four-bar mechanism to perform the transplanting motion.

#### Kinematic model simplification and analysis of the transplanting mechanism.

**Fig 3:**
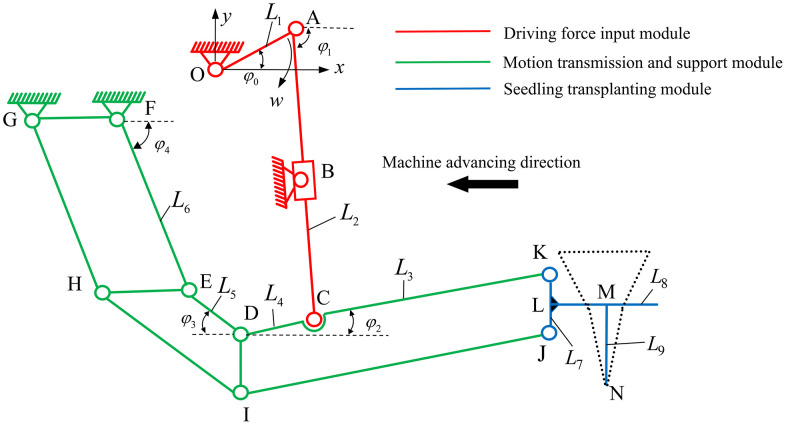
Kinematic model of planting mechanism.

In order to analyze the movement of various parts of transplanting mechanism during the transplanting process, the transplanting mechanism was simplified, as shown in Fig 3. Based on the principle of implementing functions of transplanting mechanisms, it is divided into three modules, that are driving force input module (red), motion transmission and support module (green) and seedling transplanting module (blue).

As shown in Fig 3, the driving force input module consists of crank OA, and rocker AC. The crank OA rotates about fixed pivot O and drives rocker AC, which both slides and rotates at hinge point B. The position of point B can be adjusted according to transplanting requirements. The motion transmission and support module was consisted of two parallelogram mechanisms, that are parallel four-bar mechanism Ⅰ (GFEH) and parallel four-bar mechanism Ⅱ(DIJK), which were used for supporting various mechanisms and assist in the movement of rocker AC. The seedling transplanting unit attached to parallel four-bar mechanism Ⅱ, carries a duckbill MN that remains perpendicular to the ground before entering the soil. We established a coordinate system with its origin at point O, assigning the horizontal direction as the x-axis with its positive direction opposite to the machine’s direction of travel.

Table 1 shows the description of the motion-related parameters of the adjustable nested multi-link mechanism.

**Table 1:** Motion parameter indication of planting mechanism.

Parameter	Name	Parameter	Name
*L*_1_	The length of crank OA (mm)	*φ*_1_	Angle between AC and X axis(^°^)
*L*_2_	The length of rocker AC (mm)	*φ*_2_	Angle between DC and X axis(^°^)
*L*_3_	The length of rod CK (mm)	*φ*_3_	Angle between ED and X axis(^°^)
*L*_4_	The length of rod DC (mm)	*φ*_4_	Angle between EF and X axis(^°^)
*L*_5_	The length of rod ED (mm)	*x*_B_	The x-coordinate of point B(mm)
*L*_6_	The length of rod FE (mm)	*y*_B_	The y-coordinate of point B(mm)
*L*_7_	The length of rod KJ (mm)	*x*_F_	The x-coordinate of point F(mm)
*L*_8_	Twice the length of rod LM (mm)	*y*_F_	The y-coordinate of point F(mm)
*L*_9_	The length of rod MN (mm)	*ω*	The angular velocity of the crank OA(rad·s ⁻ ¹)
*φ*_0_	Initial angle of crank OA(^°^)	*ʋ*	The velocity of the machine(mm·s ⁻ ¹)

#### Model assumption.

To facilitate the kinematic analysis and establish a solvable mathematical model, the following assumptions were made:

Rigid Link Assumption: All components of the linkage mechanism, including the cranks, rockers, and connecting rods, are considered perfectly rigid bodies. This neglects the effects of elastic deformation during operation, which is a standard and justified assumption for the structural analysis of such mechanisms given their high stiffness and the relatively low operating forces.

Ideal Joint Assumption: All revolute and sliding joints are considered ideal, meaning that clearance, friction, and wear are neglected. This allows for the precise calculation of motion transmission without considering energy loss or dynamic backlash.

Constant Forward Velocity: The transplanting machine is assumed to move forward at a constant velocity “*v*”. While minor fluctuations may occur in practice, this assumption is valid for steady-state operation on a prepared field and is essential for decoupling the mechanism’s relative motion from the machine’s absolute translation.

No Soil Interaction in Kinematic Model: The kinematic analysis presented in this section does not account for the dynamic interaction between the duckbill end-effector and the soil. The trajectory and velocity are calculated for the mechanism moving in free air. This approach is justified for two primary reasons: First, the primary goal is to design a trajectory that enables zero-speed planting prior to soil entry. Second, soil resistance is intentionally omitted during the trajectory-analysis phase because the present study focuses on the kinematic behavior of the mechanism.

These assumptions are common in the kinematic design phase of agricultural machinery and allow for the development of a tractable model that captures the essential motion characteristics

#### Motion equation of the planting mechanism.

Using the vector-loop method with the kinematic model in Fig 3, we derive the displacement, and velocity of the end-effector, aiming directly at the trajectory and velocity of the duckbill tip point N.

At time *t* = 0, the crank OA is aligned with the positive direction of the x-axis. *φ*_0_ represent the angular displacement of the crank OA at time *t* can be expressed as:


$$\varphi_{0}=-wt$$ (1)

The displacement equation for point A is:


$$\begin{array}{c} x_{A}=L_{1}\cos\varphi_{O}\\ y_{A}=L_{1}\sin\varphi_{O} \end{array}$$ (2)

Since point B is fixed, we can determine *φ*_1_ from the coordinates of A and B.:


$$\begin{array}{c} \tan\varphi_{1}=\frac{y_{A}-y_{B}}{x_{B}-x_{A}}\\ \varphi_{1}=\arctan\frac{y_{A}-y_{B}}{x_{B}-x_{A}} \end{array}$$ (3)

The constraint that point C lies on the line defined by A and B, and maintains a fixed distance *L*₂ from A, allows us to solve for its coordinates. Meanwhile, the coordinates of point C can be determined using the vector method. Thus, the position of point C can be expressed as:


$$\begin{array}{c} L_{OA}+L_{AC}=L_{FE}+L_{ED}+L_{DC}\\ x_{C}=x_{A}+L_{2}\cos\varphi_{1}\\ y_{C}=y_{A}-L_{2}\sin\varphi_{1} \end{array}$$ (4)

$$\begin{array}{c} x_{C}=x_{F}+L_{6}\cos\varphi_{4}+L_{5}\cos\varphi_{3}+L_{4}\cos\varphi_{2}\\ y_{C}=y_{F}-L_{6}\sin\varphi_{4}-L_{5}\sin\varphi_{3}+L_{4}\sin\varphi_{2} \end{array}$$ (5)

To simplify the equations, we substitute: “a” and “b” into them, resulting in the following expressions:


$$\begin{array}{c} L_{5}\cos\varphi_{3}=a,L_{5}\sin\varphi_{3}=b\\ x_{C}-x_{F}-a-L_{4}\cos\varphi_{2}=L_{6}\cos\varphi_{4}\\ y_{C}-y_{F}+b-L_{4}\sin\varphi_{2}=-L_{6}\sin\varphi_{4} \end{array}$$ (6)

Based on the functional relationship, it follows that:


$$\sin^{2}\varphi_{4}+\cos^{2}\varphi_{4}=1$$ (7)

Combining Equation (6) with Equation (7) yields:


$$[(x_{C}-x_{F}-a)-(L_{4}\cos\varphi_{2}){]}^{2}+[(y_{C}-y_{F}+b)-(L_{4}\sin\varphi_{2}){]}^{2}={L_{6}}^{2}$$ (8)

Expanding Equation (8) yields:


$$\begin{array}{c} (x_{C}-x_{F}-a{)}^{2}-2\times (x_{C}-x_{F}-a)\times L_{4}\cos\varphi_{2}+(L_{4}\cos\varphi_{2}{)}^{2}\\ +(y_{C}-y_{F}+b{)}^{2}-2\times (y_{C}-y_{F}+b)\times L_{4}\sin\varphi_{2}+(L_{4}\sin\varphi_{2}{)}^{2}={L_{6}}^{2} \end{array}$$ (9)

Based on the functional relationship, it follows that:


$$\begin{array}{c} \sin^{2}\varphi_{2}+\cos^{2}\varphi_{2}=1\\ A\cos\varphi_{2}+B\sin\varphi_{2}=C \end{array}$$ (10)

To simplify the equations, we substitute: “A”, “B” and “C” into them, resulting in the following expressions:


$$\begin{array}{c} A=2\times (x_{C}-x_{F}-a)\times L_{4}\\ B=2\times (y_{C}-y_{F}+b)\times L_{4}\\ C=(x_{C}-x_{F}-a{)}^{2}+(y_{C}-y_{F}+b{)}^{2}+L_{4}^{2}-L_{6}^{2} \end{array}$$ (11)

By combining Equations (9), (10), and (11), we can obtain *φ*₂ as:


$$\varphi_{2}=2\arctan\left(\frac{B+\sqrt{A^{2}+B^{2}-C^{2}}}{A+C}\right)$$ (12)

Based on Equation (12), the coordinates of point L are:


$$\begin{array}{c} x_{L}=x_{C}+L_{3}\cos\varphi_{2}\\ y_{L}=y_{C}+L_{3}\sin\varphi_{2}-\frac{L_{7}}{\text{2}} \end{array}$$ (13)

When the machine’s forward velocity is set to *v*, the displacement equation for the duckbill tip point N is:


$$\begin{array}{c} x_{N}=x_{L}+\frac{L_{8}}{\text{2}}-vt\\ y_{N}=y_{L}-L_{9} \end{array}$$ (14)

By combining Equations (1), (2), (3), (4), (12), (13), and (14), the equation for the coordinates of point N is obtained as:


$$\begin{array}{c} x_{N}=L_{1}\cos\varphi_{0}+L_{2}\cos\varphi_{1}+L_{3}\cos\varphi_{2}+\frac{L_{8}}{\text{2}}-vt\\ y_{N}=L_{1}\sin\varphi_{0}-L_{2}\sin\varphi_{1}+L_{3}\sin\varphi_{2}-\frac{L_{7}}{\text{2}}+\frac{L_{8}}{\text{2}} \end{array}$$ (15)

By differentiating the displacement equation of the duckbill tip point with respect to time *t*, the velocity equation for the duckbill tip point can be obtained:


$$\begin{array}{c} V_{xN}=-L_{1}w\sin\varphi_{0}-L_{2}\sin\varphi_{1}\frac{d\varphi_{1}}{dt}-L_{3}\sin\varphi_{2}\frac{d\varphi_{2}}{dt}-v\\ V_{yN}=-L_{1}w\cos\varphi_{0}-L_{2}\cos\varphi_{1}\frac{d\varphi_{1}}{dt}+L_{3}\cos\varphi_{2}\frac{d\varphi_{2}}{dt} \end{array}$$ (16)

### Influence of transplanting mechanism structural parameters on the transplanting motion characteristics

#### Software development of planting mechanism motion trajectory visualization.

**Fig 4:**
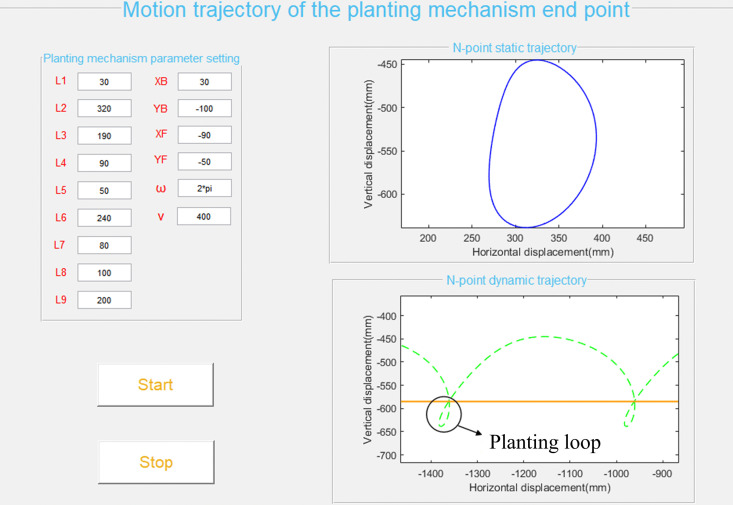
User interface of the visualization-assisted design software for planting mechanism.

Based on the established mathematical model, a dedicated Graphical User Interface (GUI) was developed in MATLAB to facilitate analysis and optimization of the adjustable nested multi-link (Fig 4). This interface is partitioned into two functional modules: parameter input and simulation results. The input panel, located on the left side of the software interface, allows for the specifications of key mechanism dimensions, while the upper right side of software interface displays the static trajectory of the duckbill tip point when machine is stationary, and the lower right side of software interface shows the dynamic trajectory of the duckbill tip point as the machine moves forward. By altering the values of various key parameters, one can change the structural parameters of the mechanism, resulting in different static and dynamic trajectory curves for the planting point.

Set the initial parameters of the transplanting machine based on experience:

*L*_1_ = 30 mm, *L*_2_ = 320 mm, *L*_3_ = 190 mm, *L*_4_ = 90 mm, *L*_5_ = 50 mm, *L*_6_ = 240 mm, *L*_7_ = 80 mm, *L*_8_ = 100 mm, *L*_9_ = 80 mm, *x*_B_ = 30 mm, *y*_B_ = −100 mm, *x*_F_ = −90 mm, *y*_F_ = −50 mm, *v = *400 mm·s ⁻ ¹, *w* = 2π.

**Fig 5:**
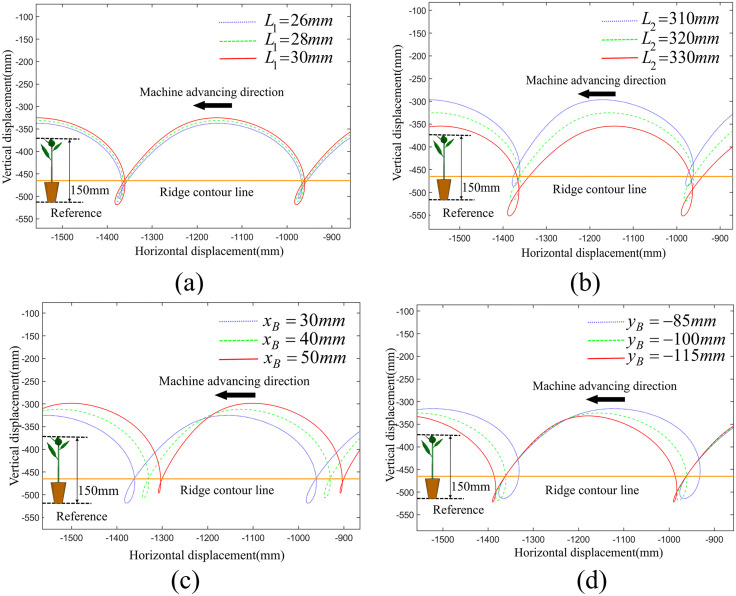
Effect of the driving force input module on planting trajectory. (a) The impact of the length of *L*_1_ on the planting trajectory, (b) The impact of the length of *L*_2_ on the planting trajectory, (c) The impact of the x-coordinate of point B on the planting trajectory, (d) The impact of the y-coordinate of point B on the planting trajectory.

**Fig 6:**
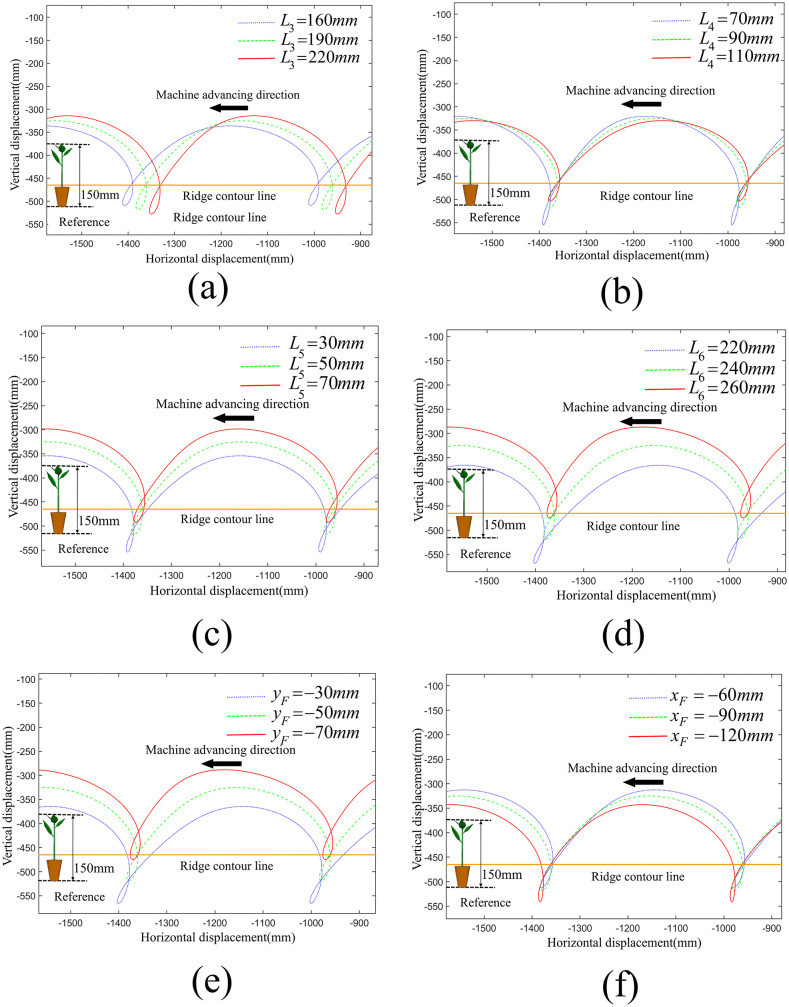
Effect of motion transmission and support module on planting trajectory. (a) The impact of the length of *L*_3_ on the planting trajectory, (b) The impact of the length of *L*_4_ on the planting trajectory, (c) The impact of the length of *L*_5_ on the planting trajectory, (d) The impact of the length of *L*_6_ on the planting trajectory, (e) The impact of the x-coordinate of point F on the planting trajectory, (f) The impact of the y-coordinate of point F on the planting trajectory.

**Fig 7:**
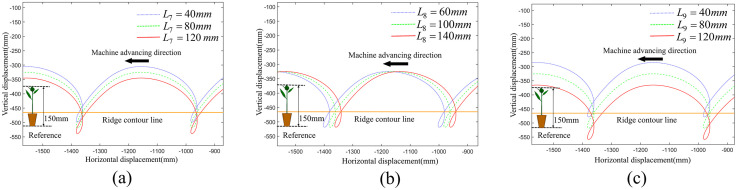
Effect of seedling transplanting module on planting trajectories. (a) The impact of the length of *L*_7_ on the planting trajectories. (b) The impact of the length of *L*_8_ on the planting trajectories. (c) The impact of the length of *L*_9_ on the planting trajectories.

We conducted a single-factor analysis on each parameter using the method of controlling variables with the developed visualization auxiliary design software. Specifically, three equally spaced values were selected for each parameter to generate the corresponding planting trajectory displacement curves of driving force input module, motion transmission and support module and seedling transplanting module respectively, as shown in Figs 5–7. Among them, the orange line represents the ridge contour line, the leftmost part of each image serves as the reference geometric height of the seedlings. When the duckbill planting end effector moves downward, the intersection point of the movement trajectory with the ridge contour line is the planting point into the soil.

#### The effect of driving force input module on the planting trajectories.

The comparative analysis of driving force input module is shown in Fig 5. Holding all other planting mechanism parameters constant, the variation in the length of crank OA(*L*_1_) exerts a significantly influence on trajectory profile, notably the total height of the planting trajectory and the depth of the planting loop. Specifically, as the length of *L*_1_ increases, both the total height of the planting trajectory and the depth of the planting loop increase,

The variation in the length of rocker AC(*L*_2_) has a slight impact on the depth and perpendicularity of the planting loop. As the length of *L*_2_ increases, the entire planting trajectory shifts downward, coupled with the increase in planting loop depth, and a degradation of its perpendicularity.

The x-coordinate of the fixed pivot B(*x*_*B*_) significantly affects the depth of the planting loop, with a slight impact on the perpendicularity of the planting loop and the total height of the planting trajectory. As *x*_*B*_ increases, the depth of the planting loop and its perpendicularity improves, while the total height of the planting trajectory decreases.

The y-coordinate of the fixed pivot B(*y*_*B*_) significantly affects the depth of the planting loop, with a slight impact on the perpendicularity of the planting loop. As the absolute value of yB increases, the depth of the planting loop decreases and its perpendicularity improves.

#### The impact of motion transmission and support module on the planting trajectories.

The comparative analysis of motion transmission and support module is shown in Fig 6. With the other parameters of the planting mechanism remaining constant, the variation in the length of link CK (*L*_3_) significantly affects the depth of the planting loop and the total height of the planting trajectory. As the length of *L*_3_ increases, the depth of the planting loop and the total height of the planting trajectory increase.

The variation in the length of link CD(*L*_4_) significantly affects the perpendicularity of the planting loop and the total height of the planting trajectory, with a slight impact on the depth of the planting loop. As the length of *L*_4_ increases, the perpendicularity of the planting loop deteriorates, and both the total height of the trajectory and the depth of the planting loop decrease.

The change in the length of link DE(*L*_5_) has a slight impact on the depth and perpendicularity of the planting loop. As the length of *L*_5_ increases, the entire planting trajectory shifts upward, the perpendicularity of the planting loop deteriorates and the depth of the planting loop increases.

The variation in the length of link EF(*L*_6_) significantly affects the total height of the planting trajectory and the perpendicularity of the planting loop, with a slight impact on the depth of the planting loop. As the length of *L*_6_ increases, the entire planting trajectory shifts upward, the perpendicularity of the planting loop improves, the depth of the planting loop increases, and the total height of the trajectory decreases.

The x-coordinate of the fixed pivot F(*x*_F_) significantly affects the perpendicularity of the planting loop, has a slight impact on the depth of the planting loop. As the absolute value of *x*_*F*_ increases, the entire planting trajectory shifts downward, the perpendicularity of the planting loop improves, and the depth of the planting loop decreases.

The y-coordinate of the fixed pivot F(*y*_F_) significantly affects the perpendicularity of the planting loop, with a slight impact on the depth of the planting loop and the total height of the planting trajectory. As the absolute value of *y*_F_ increases, the entire planting trajectory shifts upward, the perpendicularity of the planting loop improves, the depth of the planting loop increases, and the total height of the trajectory decreases.

#### The effect of seedling transplanting module on the planting trajectories.

The comparative analysis of the planting trajectory curves for seedling transplanting module is shown in Fig 7. With the other parameters of the planting mechanism remaining constant, the variation in the length of link KJ(*L*_7_) has no effect on the depth and,perpendicularity of the planting loop, as well as the total height of the planting trajectory. As the length of *L*_7_ increases, the entire planting trajectory shifts downward.

In contrast to other parameters, the variation in the length of link 2 × LM(*L*_8_) has no impact on the depth and perpendicularity of the planting loop, and the total height of the planting trajectory. As the length of *L*_8_ increases, the entire planting trajectory shifts to the right.

The variation in the length of link MN(*L*_9_) has no impact on the depth and perpendicularity of the planting loop, and the total height of the planting trajectory. As the length of *L*_9_ increases, the entire planting trajectory shifts downward.

Firstly, *L*_8_ and *L*_9_ represent the structural and mounting dimensions of the duckbill, while *L*_7_ denotes the length of the vertical member in the parallelogram linkage. The analysis indicates that these three parameters do not affect the planting trajectory, which is consistent with practical conditions. Considering the commonly used dimensions for duckbills and linkage members, the preliminary selection is made as *L*_7_ = 80 mm, *L*_8_ = 100 mm, and *L*_9_ = 200 mm.

### Structural parameters optimization of the transplanting mechanism

#### Multi-objective optimization model.

To achieve better working performance in seedling transplanting, the design requirements for the transplanting mechanism based on agronomic demands are as follows:

A. The motion trajectory of the lowest planting point entering the soil should be perpendicular to the ground.
B. The motion trajectory of the lowest planting point should not come into contact with the seedlings.
C. The planting motion trajectory should form a loop.
D. The intersection of the planting mechanism’s loop should be at the same height as the ridge surface.

Based on the above analysis, the influence of each link parameter on the motion trajectory can be determined, and the six link parameters with the greatest impact on the trajectory are identified, as shown in Table 2.

**Table 2:** The influence of each parameter on the motion trajectory.

Symbols	Key impact points	
	Significantly impact	Slight impact
*L*_1_	The perpendicularity of the planting loop、 The depth of the planting loop	None
*L*_3_	The depth of the planting loop、 The total height of the planting trajectory	None
*L*_4_	The perpendicularity of the planting loop、 The total height of the planting trajectory	The depth of the planting loop
*L*_6_	The total height of the planting trajectory、 The perpendicularity of the planting loop	The depth of the planting loop
*x*_*B*_	The depth of the planting loop	The perpendicularity of the planting loop、 The total height of the planting trajectory
*y*_*B*_	The depth of the planting loop	The perpendicularity of the planting loop
*x*_*F*_	The perpendicularity of the planting loop	None
*y*_*F*_	The perpendicularity of the planting loop	The depth of the planting loop、 The total height of the planting trajectory

Given the large number of design variables and the mutual constraints among competing objectives, traditional single-objective methods are prone to trapping in local optima under strongly constrained and highly coupled conditions. Therefore, a multi-objective genetic algorithm was adopted to simultaneously capture the entire Pareto frontier of optimal trade-off solutions, ensuring both global exploration of the parameter space and robust convergence [27].

This study employs a multi-objective genetic algorithm to optimize eight key parameters of the planting mechanism, with the following configurations: PopulationSize = 100, MaxGenerations = 150, and ParetoFraction = 0.35. The parameters to be optimized range as follows: *L*_1_ (26 mm to 30 mm), *L*_3_ (160 mm to 220 mm), *L*_4_ (70 mm to 110 mm), *L*_6_ (220 mm to 260 mm), *x*_B_ (30 mm to 50 mm), *y*_B_ (−115 mm to −85 mm), *x*_F_ (−120 mm to −60 mm), and *y*_F_ (−70 mm to −30 mm). All eight parameters were explicitly defined as integer variables during the optimization process to ensure a manufacturability and assembly precision. The optimization objectives are to minimize the deviation of the planting loop depth from the target value of 60 mm and the deviation of the planting loop perpendicularity from the target value of 90°, while constraining the total trajectory height to be no less than 150 mm. The trajectory is calculated based on a kinematic model, and loop-forming features are automatically identified using a line intersection method. Finally, a comprehensive optimal solution is selected from the Pareto front. The corresponding trajectory satisfies the design requirements by achieving a forming distance close to 60 mm, a forming angle close to 90°, and a total height greater than 150 mm.

At the set transplanting velocity of 400 mm·s ⁻ ¹, an optimal analysis of the planting mechanism is conducted. Through multi-objective genetic algorithm, an optimal set of parameters is ultimately obtained:

*L*_1_ = 30 mm, *L*_2_ = 320 mm, *L*_3_ = 206 mm, *L*_4_ = 72 mm, *L*_5_ = 50 mm, *L*_6_ = 245 mm, *L*_7_ = 80 mm, *L*_8_ = 100 mm, *L*_9_ = 200 mm, *x*_B_ = 50 mm, *y*_B_ = −94 mm, *x*_F_ = −80 mm, *y*_F_ = −64 mm, *v = *400 mm·s ⁻ ¹, *w* = 2π.

**Fig 8:**
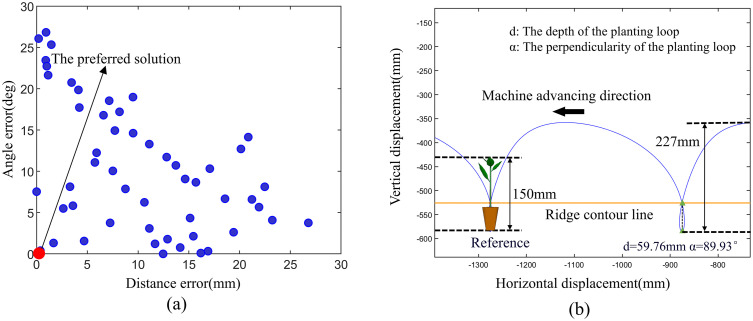
Multi-objective optimization results and the corresponding optimal trajectory. (a) The Pareto front of the optimization objectives, (b) The Optimized kinematic trajectory.

As shown in Fig 8, the Pareto front Fig 8(a) illustrates the trade-off between the two objectives, while the optimized kinematic trajectory Fig 8(b) of the selected point demonstrates that the optimal design successfully meets the agronomic requirements, the perpendicularity of the planting loop is 89.93°, and the depth of the planting loop is 59.76 mm. The motion trajectory of the lowest planting point entering the soil is perpendicular to the ground, ensuring the uprightness of the seedlings after transplanting. The maximum height of the seedlings is 150 mm, and the total height of the planting trajectory is 227 mm, which ensures that the motion trajectory does not come into contact with the seedlings. The depth of trays on the market is generally 40 mm, the planting depth is generally slightly deeper than the pot diameter, which is the height about 60 mm. The planting motion trajectory forms a loop below the soil surface, creating zero-speed transplanting. The intersection of the planting mechanism’s loop is consistent with the height of the ridge surface, ensuring that the zero-speed point is in the soil and guaranteeing planting quality. The upper curve of the planting motion trajectory is gentle, which can increase the success rate of seedling placement.

**Fig 9:**
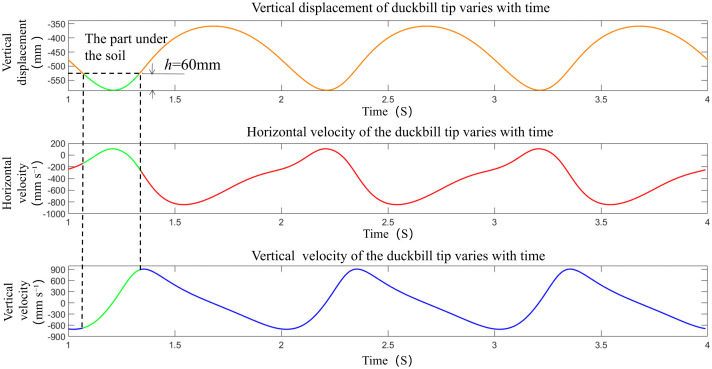
The comparison of velocity and displacement at the duckbill tip.

Based on the optimal parameters, MATLAB was used to plot the velocity change graphs of the duckbill tip point in both horizontal and vertical directions, and the duckbill’s motion velocity was analyzed with reference to the vertical displacement. As shown in Fig 9, the coordinates of the lowest point of the vertical displacement of the duckbill tip are −585 mm, and the coordinates of the highest point are −358 mm, with a height difference of 227 mm. This is higher than the seedlings height, which meets the actual planting requirements. After the duckbill tip enters the soil, its horizontal component of velocity first approaches 0, then increases to about 106 mm·s ⁻ ¹ in the positive direction, decreases to 0, and finally decreases to about −280 mm·s ⁻ ¹ in the negative direction. There are two points where the horizontal component of velocity is 0 after the seedling enters the soil.

This is because the trajectory of the duckbill tip forms a loop, and the horizontal component of velocity is equal in magnitude but opposite in direction to the motion velocity of the transplanting machine, achieving zero-speed planting, which improves the uprightness of planting. When the planting mechanism picks up the seedlings near the highest point, the horizontal seedling-picking speed decreases, and the vertical seedling-picking speed approaches zero. This reduces the impact on the seedlings and ensures the quality of seedling-picking. According to the vertical velocity variation over time, the vertical velocity at the lowest planting point is 0, which is consistent with the actual situation.

#### Planting performance analysis under different spacing.

Based on the optimized parameters obtained, the structural design of each component is carried out. As previously analyzed, the vertical coordinate of the fixed pivot point B (rotation center in the power transmission module) can be adjusted by moving the planting trajectory adjustment mechanism up and down, which drives the regulator rod to adjust the vertical coordinate of rotation center in the power transmission module, thereby changing the transplanting trajectories. The planting depth can be adjusted by repositioning the adjustment rod to suit different planting needs.

**Fig 10:**
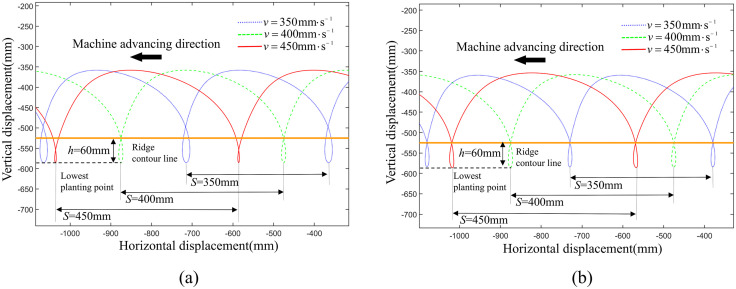
The transplanting trajectories with different planting spacing before and after parameters optimization. (a) transplanting trajectories before parameters optimization; (b) transplanting trajectories after parameters optimization.

Here, “S” represents the plant spacing and “*h*” represents the required planting depth. To verify the planting effect of the planting mechanism at different plant spacing, the change in planting trajectories were observed by only altering the travel velocity of the transplanting machine while keeping the planting frequency at 60 plants per minute. As can be seen from Fig 10(a), the planting spacing were 350 mm, 400 mm, and 450 mm, respectively. When the planting spacing was 400 mm, the depth of the planting loop was 60 mm, with the entry point at the loop intersection and the zero-speed point located below the soil surface. However, when the actual planting spacing were 350 mm and 450 mm, the loop in the trajectory gradually changed, leading to a deterioration in planting effectiveness.

From previous analysis, it is known that decreasing the absolute value of *y*_*B*_ can increase he depth of the planting loop, therefore, as shown in Fig 10(b), with all other parameters remaining constant, when the planting spacing is 350 mm, the *y*_*B*_ coordinate is adjusted to −105 mm, and when the planting spacing is 450 mm, the *y*_*B*_ coordinate is adjusted to −83 mm, which can maintain the final planting effect. Thus, by adjusting the y-coordinate of the fixed pivot point B, the loop in the planting trajectory can be kept constant as the planting spacing increases, thereby ensuring that the planting effect remains unchanged.

## Results and discussions

### Experimental verification of the planting mechanism trajectories

**Fig 11:**
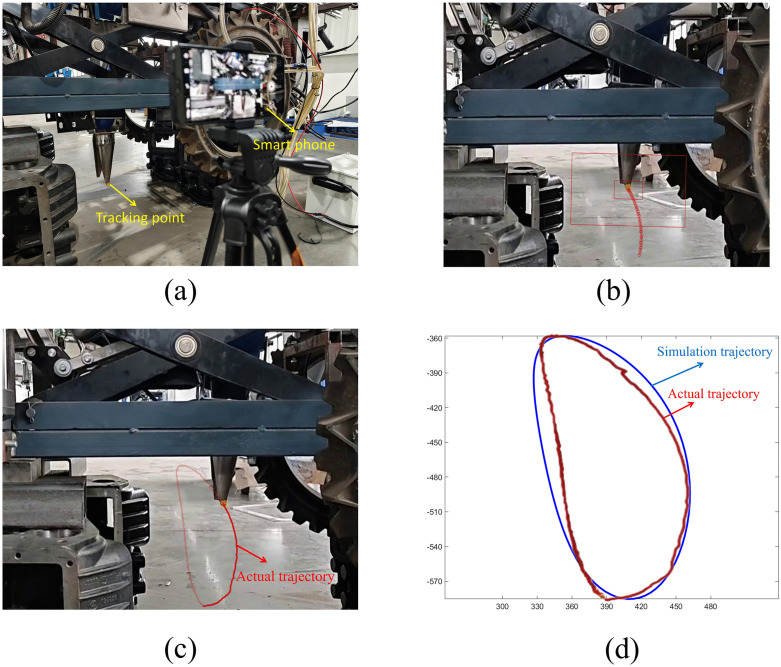
Simulation static trajectory of the planting mechanism. (a) Trajectory verification test bench, (b) Trajectory tracking of the tip of the duckbill (c) Actual trajectory of the tip of the duckbill; (d) Observation between the simulated and experimental trajectory results.

To validate the rationality of the mechanism design, we conducted a simulation analysis using MATLAB software. We set the planting spacing at 400 mm, and the planting frequency at 60 plants per minute. The experimental validation test rig configuration is shown in Fig 11(a).

To improve the verifiability of motion trajectory analysis, this study employed a visual marking method to label the terminal component of the duckbill transplant machine. The operational protocol consisted of: 1) attaching brown marker tape to the surface of the gripper’s end-effector as a tracking reference point, which amplified chromatic contrast between target features and background environment, thereby enhancing feature recognition accuracy during trajectory tracking; capturing motion data through smartphone videography; extracting trajectory coordinates using the open-source tracking software Kinovea [28–30].

Fig 11(b) displays the trajectory tracking process, while Fig 11(c) illustrates the actual trajectory. A comparative analysis of consistency between simulated trajectories and experimental measurements is presented in Fig 11(d). Fig 11(d) shows that the actual trajectory largely coincides with the simulated trajectory, with a maximum error not exceeding 15 mm. The possible reasons for the error may include mechanical installation accuracy and mechanical vibration.

### Field testing and verification of the planting mechanism

**Fig 12:**
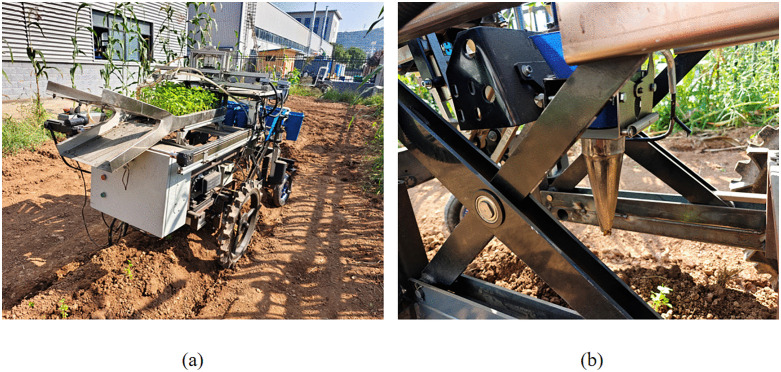
Filed transplanting experiment. (a) Overall equipment operation, (b) Planting Device Operation.

To verify the transplanting effect of the planting mechanism after parameter optimization, a adjustable nested multi-link mechanism planting test machine was manufactured based on the optimized parameters, and the field test was conducted with the seedling upright degree as the main inspection index. The production of this machine was carried out at Baoji Dingduo Machinery Co., Ltd. in Shanxi, and the test transplanting machine is shown in Fig 12(a) and 12(b).

To ensure suitable transplanting operations, preparatory work such as tilling was completed beforehand. The ridge width was 600 mm, and the ridge height was 200 mm,The soil moisture content was maintained at 17–18% through controlled irrigation prior to testing, which corresponds to the optimal range for celery transplanting operations in dryland conditions. This moisture level ensures adequate soil cohesion for stable hole formation by the duckbill planter while promoting proper root-soil contact after transplanting. The selected celery seedlings (*Apium graveolens*) had an average height of 124 mm with a standard deviation of ±6.8 mm, indicating good uniformity within the test population. This height range (115–130 mm) represents the typical specification for celery transplanting in the Baoji region and seedlings of this size possess sufficient stem mechanical strength to maintain posture during mechanical handling.

To verify the transplanting effect of the optimized planting mechanism, a physical prototype of the adjustable nested multi-link planting mechanism, based on the optimized structural parameters, was constructed. The transplanting effect of the prototype was validated through field tests in accordance with the standard file for dry land planting machine (JB/T 10291–2013, Transplanter of dry land plant) [31]. This standard file requires the lodging planting rate of less than 7%, the missing planting rate of less than 5%, the coefficient of variation of plant spacing of less than 15%, the planting depth qualification rate of greater than 75%, and the planting qualification rate of greater than 90%. The formulas for the performance indicators calculation of transplanting are as follows.

The lodging planting rate (*T*):


$$T=\frac{N_{DF}}{N^{\prime }}\times 100\%$$ (17)

Where *N*_DF_ represents the number of lodged planting seedlings, *N’* represents the theoretical number of seedlings in the testing section. The definition of lodging seedlings is that after planting, the angle between the stem of the seedling and the horizontal ground is less than 30°.

The missing planting rate (*E*):


$$E=\frac{N_{LZ}}{N^{\prime }}\times 100\%$$ (18)

Where *N*_LZ_ represents the number of missed planting seedlings.

The missing planting rate (*CV*_X_):


$$CV_{X}=\frac{S_{X}}{\overset{―}{X}}\times 100\%$$ (19)

Where *S*_X_ represents the standard deviation of plant spacing, *$\overset{―}{X}$* represents the mean value of plant spacing.

The planting depth qualification rate (*H*):


$$H=\frac{N_{h}}{N^{\prime }}\times 100\%$$ (20)

Where *N*_*h*_ represents the seedlings number of plants with qualified planting depth. The qualified planting depth means standard depth is *h* cm, and the qualified depth is $h_{-1}^{\text{2}}$ cm. The standard planting depth set in this study is 6 cm, so the qualified planting depth is 5–8 cm.

The planting qualification rate (*Q*):


$$Q=\frac{N_{HG}}{N}\times 100\%$$ (21)

Where *N*_HG_ represents the seedlings total number of planted seedlings without over planting, missing planting, lodging planting, buried seedlings, or damaged seedlings, *N* represents the total number of seedlings in the testing section.

To accommodate different plant spacing requirements, a comparative analysis of planting performance was conducted before and after the structural parameter optimization. The transplanting spacing was set to 350 mm, 400 mm and 450 mm, with a planting frequency of 60 seedlings per minute. Accordingly, the forward speed of the transplanter was set at 350 mm·s^-1^, 400 mm·s^-1^, and 450 mm·s^-1^. respectively. For each plant spacing, the experiment was repeated five times with 30 seedlings per replicate.The testing results are shown in Table 3. Data are presented as mean ± standard deviation. T-tests were performed comparing overall performance before and after adjustment across all plant spacings (350, 400, and 450 mm). “*” represent p < 0.05, “**” represent p < 0.01.

**Table 3:** Main transplanting performance before and after structural parameters optimized adjustment.

Target	Lodging Rate(%)			Missing planting rate(%)			Coefficient of variation of plant spacing(%)			Planting depth qualification rate(%)			Planting qualification rate(%)		
Design plant spacing(mm)	350	400	450	350	400	450	350	400	450	350	400	450	350	400	450
Before structural parameters adjustment	0.67 ± 1.49	4.00 ± 2.79	4.67 ± 1.83	0.00 ± 0.00	0.00 ± 0.00	1.33 ± 1.83	4.06 ± 0.36	2.79 ± 0.11	2.68 ± 0.13	95.33 ± 1.83	95.33 ± 3.33	90.67 ± 7.23	95.33 ± 1.83	94.67 ± 2.98	93.33 ± 3.03
After structural parameters adjustment	0.00 ± 0.00	2.00 ± 1.83	1.33 ± 1.83	0.00 ± 0.00	0.00 ± 0.00	0.67 ± 1.49	3.73 ± 0.21	2.76 ± 0.16	2.54 ± 0.19	98.67 ± 1.83	92.67 ± 4.94	94.00 ± 4.94	98.67 ± 1.83	97.33 ± 1.49	98.00 ± 1.83
T-value	2.55			0.56			2.84			−1.87			−4.67		
p-value	0.022			0.58			0.109			0.082			0.0003		
Significance	*												**		

Fig 12 demonstrates that the planting mechanism follows an oval trajectory. Notably, the trajectories obtained from theoretical analysis, simulation, and the physical prototype are highly consistent. Minor deviations observed in the experimental trajectory are likely due to mechanical vibrations and assembly tolerances. This consistency confirms that the actual mechanism achieves zero-speed transplanting at the soil entry point, as designed in the simulation, which is crucial for ensuring transplanting quality.

The field performance data summarized in Table 3, validate the effectiveness of the structural parameter optimization. The qualified transplanting rate reached 98% after optimization, which is 3.56% higher than the pre-optimization rate. Furthermore, all other key performance indicators met the standard requirements, including a lodging rate of 1.11%, a missing planting rate of 0.22%, a plant spacing variation coefficient of 3.01%, and a planting depth qualification rate of 95.11%. These results conclusively demonstrate that the optimized nested multi-link mechanism delivers reliable performance, supported by a trajectory that enables zero-speed planting.

In comparison with prevailing techniques, the proposed mechanism operating at plant spacings of 350 mm, 400 mm and 450 mm achieves a mean qualification rate of 98% together with an exceptionally low lodging rate. These performance levels match or exceed recently reported results for multi-link or duckbill planters under comparable field conditions. Such improvement originates from the optimized trajectory, which brings the duckbill tip to a stable, near-zero velocity immediately beneath the ridge surface. This allows the seedling to be released into the soil with virtually no dynamic disturbance, ensuring high uprightness and a minimal miss rate.

Despite the significant improvements, some transplanting failures still occurred. The preliminary observations indicated that the uneven ridge height and poor surface quality resulting from inadequate land preparation were the primary factors affecting seedling erectness and the missing rate. Therefore, Improving land leveling quality is essential for further enhancing the transplanting success rate. Moreover, the applicability of the current optimization method may be influenced by specific soil conditions (e.g., clay-heavy or sandy soils that affect duckbill penetration and closure) and seedling parameters (e.g., pot size or root strength). Future work should systematically evaluate the mechanism’s performance across these varying conditions to further generalize and refine the optimization framework.

Additionally, the planting qualification rate exhibited a slight decrease with increased plant spacing. This is because a larger planting distance requires a higher forward speed at a constant planting frequency, which increases the dynamic forces acting on the seedlings and can lead to leakage or lodging. To mitigate this, further optimization of the duckbill structure is planned. A key focus will be the use of Discrete Element Method (DEM) simulations to gain deeper insights into the soil dynamics during duckbill-soil interaction. This approach will help clarify how soil flow and compaction around the duckbill influence seedling release and uprightness, providing a theoretical basis for structural optimization aimed at minimize disturbance and enhance planting stability. Additionally, the use of wear-resistant or low-friction materials will be explored to enhance durability.

Finally, it should be noted that although the sample size of 150 seedlings per group in this study provides solid evidence for the mechanism’s performance, future research could further enhance the statistical robustness and generalizability of the findings by including more replications across multiple growing seasons and locations. Likewise, the mechanism’s long-term durability and energy consumption are key considerations for commercialization, necessitating future work on wear testing and energy optimization to ensure cost-effectiveness.

## Conclusions

An adjustable nested multi-link mechanism was designed, and a mathematical model of this mechanism was established. By combining the kinematic model and developing a MATLAB/GUI for human-computer interaction, the influence patterns of the main parameters on the duckbill’s motion trajectory were derived, resulting in structural parameters that meet the requirements for seedling planting operations.

At a transplanting spacing of 400 mm, a set of optimal link parameters was selected according to agronomic requirements. The resulting motion trajectory is perpendicular to the ground, meeting the requirements for uprightness. The motion trajectory curve does not touch the seedlings. An analysis of the horizontal velocity at the duckbill tip indicates that it meets the zero-speed planting requirement after entering the soil.

The transplanting machine adjusts the coordinates of the fixed pivot point B to accommodate the planting requirements for seedlings with various plant spacing. At a planting frequency of 60 plants per minute, simulation analysis was conducted on the planting trajectories for plant spacing of 350 mm, 400 mm, and 450 mm. The results indicated that by regulating the y-coordinate of the fixed pivot point B, both the uprightness of the plants and the planting effectiveness can be ensured.

By comparing the simulation results with the actual trajectory, it can be concluded that the endpoint trajectory of the planting mechanism closely matches simulation, and forming an oval shape.

The field test results show that, at a planting frequency of 60 plants per minute, the transplanter demonstrates excellent planting performance at transplanting spacings of 350 mm, 400 mm, and 450 mm. The findings of this study provide valuable guidance for the optimized design of a multi-link planting mechanism to improve planting quality.
